# The Medical Aspects of Carcinoma of the Breast

**Published:** 1906-02-03

**Authors:** 


### THE MEDICAL ASPECTS OF CARCINOMA OF THE BREAST.

In an address on this subject, Professor Wm. Osler deals *inter alia* with the question of secondary developments in the form of metastases. He groups these under the headings—intrathoracic, abdominal, cerebral, spinal, and skeletal. In connection with the first he notes extension through the chest wall to the pleura, with secondary involvement of the lymphatic glands, and, less commonly, disease of the lung itself, as frequent consequences of mammary carcinoma. Pleurisy with effusion may come on insidiously, and the only symptom may be shortness of breath; in other cases there are severe pains. Intrathoracic glandular metastases are very common, and tend to produce all the pressure symptoms of tumour; the glands above the clavicle may be enlarged. Involvement of the lung as a consequence of breast cancer, at least to such an extent as to produce conspicuous symptoms, is uncommon. Among abdominal complications direct extension to the peritoneum and a recurring ascites must be recognised. These may even be the prominent clinical facts, the breast tumour being latent or its existence being concealed. Large lymphatic tumours may be formed in the lymphatic glands and omentum. Metastasis to the liver is frequent, but it is not always associated with symptoms and thus often possesses post-mortem rather than clinical interest.

Cerebral metastases include secondary developments both in the bones of the skull and in the brain itself. Among the resulting symptoms may be noted headache, disturbances of vision, ataxia, paralyses, optic neuritis, etc. These developments may follow years after the removal of the primary tumour, or may occur in a patient with an old atrophic scirrhus. A case is quoted of a patient, aged 68, who suddenly developed Jacksonian epilepsy, with subsequent hemiplegia and coma as a result of metastasis to the brain, secondary to a malignant tumour of the breast which had been operated on seven years previously. In a similar fashion a monoplegia, a hemiplegia, or a convulsive seizure may be the first evidence of a more or less remote cerebral complication of carcinoma of the breast.

Secondary lesions in the spinal cord are common, and are serious as entailing a maximum of suffering. Sever paroxysms of pain, and paraplegia, may result from tumours, even of very small size. Here, again, the primary event may be the atrophic form of scirrhus of the breast, and may thus be overlooked, the case coming before the physician as one of disease of the spinal cord. It is rare to see kyphosis. In some instances the secondary growths may become sclerotic and shrink, and thus there may be disappearance of the symptoms of pressure on the cord. The symptoms usually occur in two stages. In the first or neuralgic stage—perhaps a few months to two years after the removal of the breast—there are pains in the back and side, often indefinite in character and ill-defined as to position, and apt, in the absence of impairment of the general health, to be regarded as evidences of neurasthenia or hysteria. With these pains there is an exalted sensibility, especially to jarring movements. The symptoms may persist for months without the slightest sign of organic change, and thus the physician may be much puzzled as to their interpretation. Sometimes there is an outbreak of shingles. Gradually the pains become more severe, and while, as a rule, thoracic or lumbar may for a time be entirely sciatic, and may affect one or both limbs. With all this there may be no disturbances either of sensation or motion. The reflexes are often exaggerated, but not more than is common in neurasthenia. The second stage is a pressure paraplegia, usually of the spastic type. The course is usually slow, but occasionally paralysis may be complete in the course of a few days. Cramp or painful spasms are common, and finally there is the well-known picture of paraplegia dolorosa. In rare cases only one leg is paralysed. It is a remarkable thing that the secondary tumours sometimes undergo spontaneous involution, with corresponding relief to the symptoms. Thus paraplegia and other disturbances may be considerably modified.

Metastasis to the bones is common, and indeed in many of the cases with brain and spinal cord symptoms these depend on secondary growths in the skull and vertebræ. The appearance of a tumour or a spontaneous fracture may be the first indication of a growth in one of the long bones. Sometimes there is a widespread generalisation.

It may be hoped that the radical operation as now adopted will, if performed early, lessen the number of these distressing cases with internal metastases. When the development has actually occurred the only treatment is morphine in sufficiently large doses to relieve the patient's sufferings.

